# Rational Construction of Hierarchically Porous Fe–Co/N-Doped Carbon/rGO Composites for Broadband Microwave Absorption

**DOI:** 10.1007/s40820-019-0307-8

**Published:** 2019-09-13

**Authors:** Shanshan Wang, Yingchun Xu, Ruru Fu, Huanhuan Zhu, Qingze Jiao, Tongying Feng, Caihong Feng, Daxin Shi, Hansheng Li, Yun Zhao

**Affiliations:** 1 School of Chemistry and Chemical Engineering, Beijing Institute of Technology, Zhuhai, 100081 People’s Republic of China; 2 School of Materials and the Environment, Beijing Institute of Technology, Zhuhai, 519085 People’s Republic of China

**Keywords:** Fe-doped Co-MOF, Hierarchically porous, rGO, Broadband, Microwave absorption performance

## Abstract

Developing lightweight and broadband microwave absorbers for dealing with serious electromagnetic radiation pollution is a great challenge. Here, a novel Fe–Co/N-doped carbon/reduced graphene oxide (Fe–Co/NC/rGO) composite with hierarchically porous structure was designed and synthetized by in situ growth of Fe-doped Co-based metal organic frameworks (Co-MOF) on the sheets of porous cocoon-like rGO followed by calcination. The Fe–Co/NC composites are homogeneously distributed on the sheets of porous rGO. The Fe–Co/NC/rGO composite with multiple components (Fe/Co/NC/rGO) causes magnetic loss, dielectric loss, resistance loss, interfacial polarization, and good impedance matching. The hierarchically porous structure of the Fe–Co/NC/rGO enhances the multiple reflections and scattering of microwaves. Compared with the Co/NC and Fe–Co/NC, the hierarchically porous Fe–Co/NC/rGO composite exhibits much better microwave absorption performances due to the rational composition and porous structural design. Its minimum reflection loss (RL_min_) reaches − 43.26 dB at 11.28 GHz with a thickness of 2.5 mm, and the effective absorption frequency (RL ≤ − 10 dB) is up to 9.12 GHz (8.88–18 GHz) with the same thickness of 2.5 mm. Moreover, the widest effective bandwidth of 9.29 GHz occurs at a thickness of 2.63 mm. This work provides a lightweight and broadband microwave absorbing material while offering a new idea to design excellent microwave absorbers with multicomponent and hierarchically porous structures.

**Electronic supplementary material:**

The online version of this article (10.1007/s40820-019-0307-8) contains supplementary material, which is available to authorized users.

## Highlights

- Hierarchically porous Fe–Co/N-doped carbon/rGO (Fe–Co/NC/rGO) composites were successfully prepared. Macropores, mesopores, and micropores coexisted in the composites.
- Hierarchically porous Fe–Co/NC/rGO showed effective bandwidth of 9.29 GHz.

**Electronic supplementary material:**

The online version of this article (10.1007/s40820-019-0307-8) contains supplementary material, which is available to authorized users.

## Graphical abstract

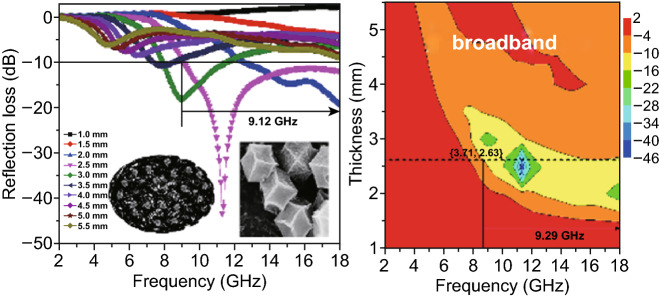

## Introduction

With the rapid popularization of electronic equipment, electromagnetic radiation pollution brings disturbance to the proper functioning of electronic equipment as well as significant dangers to military security and human health [1–4]. Therefore, it is critical to develop efficient microwave absorbing materials that are lightweight and thin with strong broadband absorption to address this serious problem [5–7]. According to the microwave absorption mechanism, when absorbers have magnetic loss, dielectric loss, and impedance match, more microwaves can enter the interior of the materials and be converted into other forms of energy and dissipated [8, 9]. In past decades, many efforts were devoted to researching composites for enhancing microwave absorption, such as (Fe, Ni, Co)/C [10–12], (Fe, Ni, Co)/rGO [13–15], MFe_2_O_4_/rGO (M = Fe, Ni, Co) [16–19], MFe_2_O_4_/CNTs [20–22], and NiO/SiO_2_/Fe_3_O_4_/rGO [23, 24]. However, these materials have the shortcoming of narrow absorption bands, which seriously hinders their practical applications.

Recently, metal–organic frameworks (MOFs) have attracted wide attention due to their unique structures [25, 26]. The Co-MOF-derived Co/C composites have the advantages of low density, nanoporous structure, multiple polarization centers, and the coexistence of magnetic loss and dielectric loss, which are beneficial to improve the impedance matching and multiple reflection and scattering of microwaves. Zhu et al. synthesized Co/C composites by carbonization of Co-MOFs; the minimum reflection loss (RL_min_) of the composites was − 15.7 dB, and the effective bandwidth was 5.4 GHz (12.3–17.7 GHz) at a thickness of 1.7 mm [27]. Wang et al. fabricated Co–C composite using Co-MOF-74 as the precursor; its RL_min_ reached − 62.12 dB, while its effective bandwidth was 4.6 GHz (10.1–14.7 GHz) at the thickness of 2.4 mm [28]. Liao et al. prepared Co/ZnO/C absorbers from cuboid-shaped heterobimetallic MOFs; the optimized RL_min_ was − 52.6 dB, and the effective bandwidth was 4.9 GHz at a thickness of 3.0 mm [29]. Wang et al. reported a Co nanoparticles (NPs)/porous C composite by annealing Co NPs/ZIF-67; its RL_min_ was − 30.31 dB, and the effective bandwidth was 4.93 GHz [30]. Although Co-MOF-based Co/C composites show good microwave absorption performances, their narrow absorption bands still need to be improved. Thus, in order to solve the problem of the impedance mismatch of Co/C composites derived from weak magnetic loss of single Co-MOF and further improve absorbers’ microwave absorption performance, we designed porous Fe–Co/N-doped C (Fe–Co/NC) composites derived from Fe-doped Co-MOF by doping strongly magnetic Fe into Co-MOF. At the same time, NC obtained by the carbonization of Fe-dope Co-MOF is of great benefit to enhance the dielectric loss.

In addition, rGO with a porous structure can effectively broaden the effective bandwidth because the microwaves entering the pores of rGO can be reflected and multiply scattered [31–36]. Hence, we introduce the Fe-doped Co-MOF into porous cocoon-like rGO to prepare multicomponent and hierarchically porous Fe–Co/NC/rGO composites. In this way, the multicomponent materials can possess both magnetic loss and dielectric loss and achieve impedance matching to enhance the absorbing performance. At the same time, the hierarchically porous structures of the materials can reduce the density and broaden the effective bandwidth to obtain lightweight absorbers with strong broadband absorption.

Herein, hierarchically porous Fe–Co/NC/rGO composites were designed and prepared by carbonization of Fe-doped Co-MOF grown in situ on the porous cocoon-like rGO. The Fe–Co/NC/rGO with multiple components and unique hierarchically porous structures exhibited the widest effective bandwidth of 9.29 GHz at a thickness of 2.63 mm. Compared to Co/NC and Fe–Co/NC, the absorbing mechanism of Fe–Co/NC/rGO was explained. This design strategy of multicomponent and hierarchically porous structures provides a new research direction for the development of lightweight and broadband microwave absorbing materials.

## Experimental

### Materials

Cobalt nitrate hexahydrate (Co(NO_3_)_2_·6H_2_O), ferrous sulfate heptahydrate (FeSO_4_·7H_2_O), 2-methylimidazole (2-MIM), ascorbic acid (VC), and methanol were purchased from Beijing Chemicals and used without further purification.

### Synthesis of Hierarchically Porous Fe–Co/NC/rGO

The hierarchically porous Fe–Co/NC/rGO composite was synthesized using the following steps. First, GO was prepared via the modified Hummers method [37]. Second, porous cocoon-like rGO was fabricated using our previously reported method [33] (SI). Third, 15 mg of porous cocoon-like rGO was dispersed in 10 mL of methanol and marked as solution A. 0.4514 g of 2-MIM and 0.3637 g of Co(NO_3_)_2_·6H_2_O were separately dissolved in 10 mL of methanol. They were uniformly mixed under stirring and marked as solution B. 0.037 g of FeSO_4_·7H_2_O was dissolved in 10 mL of methanol and mixed with solution B to form a uniform solution. Sequentially, solution A was added into the above mixed solution and stirred for 30 min continuously. After the mixture was aged at 25 °C for 24 h, the products were collected by centrifugation and washed with deionized water. The Fe-doped Co-MOF/rGO was obtained by freeze drying. Then, Fe-doped Co-MOF/rGO was heated at a rate of 5 °C min^−1^ and calcined at 600 °C in Ar for 2 h to obtain hierarchically porous NC/rGO composites embedded with Co_3_Fe_7_ and Co (Fe–Co/NC/rGO).

For comparison, Co/NC was prepared by the same process without solution A and a methanol solution of FeSO_4_·7H_2_O. Fe–Co/NC was prepared using the same process without solution A.

**Fig. 1:**
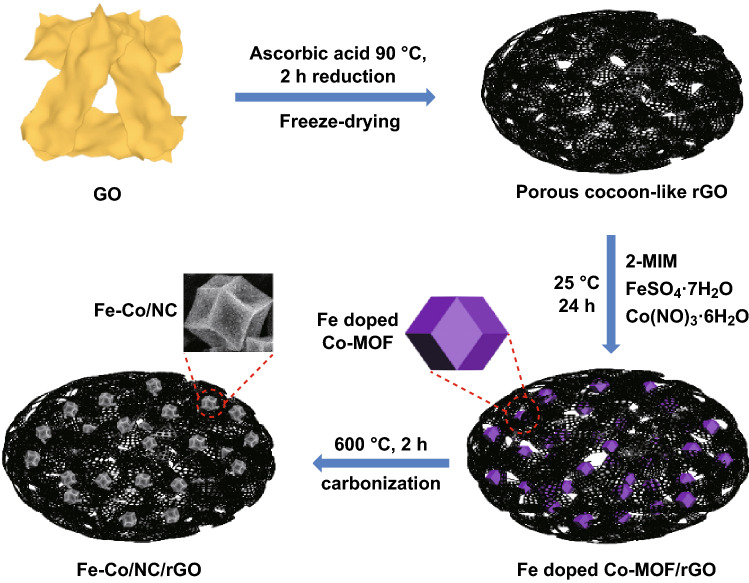
Schematic drawings illustrating fabrication process of hierarchically porous Fe–Co/NC/rGO

The diagram of the preparation procedure for Fe–Co/NC/rGO is shown in Fig. 1.

### Characterization

The morphologies and size of the samples were analyzed by a field emission scanning electron microscope (FESEM; JEOL JSM-7500F) and high-resolution transmission electron microscope (HRTEM; Hitachi HT7700). The crystal structures of the samples were detected by X-ray diffraction (XRD; UItima IV, 40 kV, 150 mA, Cu Kα radiation). The structural characteristics of carbon materials were characterized by a Raman spectrometer (LabRAM ARAMIS; λ = 514 nm). The element compositions and chemical binding states of the samples were determined by X-ray photoelectron spectroscopy (XPS; Thermo ESCALAB 250XI). The pore size distribution of the samples was measured using a specific surface area and pore structure analyzer (Micrometrics ASAP 2460) and analyzed by the Brunauer–Emmett–Teller (BET) method. The magnetic properties of the samples were tested by a vibrating sample magnetometer (VSM; Lakeshore, model 7404 series). The electromagnetic parameters were measured by a vector network analyzer (PNA-N5244A; Agilent coaxial method) in the range of 1–18 GHz. The preparation process of coaxial rings for electromagnetic parameter measurement is shown in Fig. S1.

## Results and Discussion

### Morphologies and Structures

**Fig. 2:**
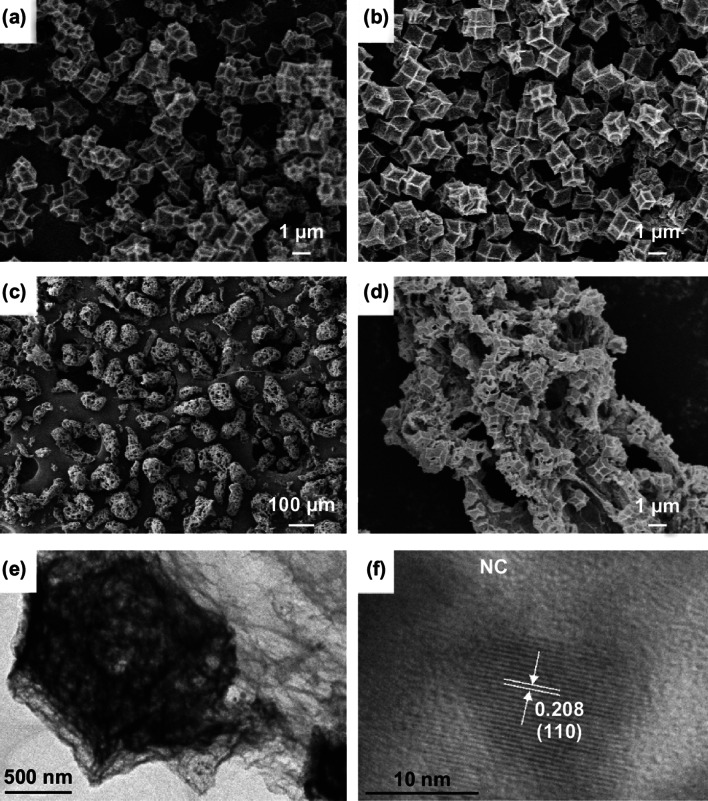
FESEM images of **a** Co/NC, **b** Fe–Co/NC, **c**, **d** Fe–Co/NC/rGO, and **e**, **f** HRTEM images of the Fe–Co/NC/rGO

As exhibited in Fig. S2a, b, Co-MOF shows a smooth and complete dodecahedral morphology; its average particle size is approximately 800 nm. Fe-doped Co-MOF also presents the same morphology, while the average particle size is larger than that of Co-MOF, which is approximately 1.3 μm. The SEM images of Co/NC, Fe–Co/NC, and Fe–Co/NC/rGO are displayed in Fig. 2. Compared with Co-MOF and Fe-doped Co-MOF, Co/NC and Fe–Co/NC (Fig. 2a, b) obtained by the high-temperature carbonization of Co-MOF and Fe-doped Co-MOF can basically maintain the morphologies of their corresponding MOF precursors, and their skeletal structure shrinks slightly. The surfaces of the samples become rough and sunken. As shown in Fig. S2c, cocoon-like rGO with a porous structure is obtained by the simple and green reduction method. In Fig. 2c, after the in situ growth of Fe-doped Co-MOF on rGO and carbonization, Fe–Co alloy embedded porous NC (Fe–Co/NC) composites are uniformly distributed on the porous rGO sheets. The Fe–Co/NC/rGO shows a hierarchically porous structure. In Fig. S2d, the Fe–Co/NC/rGO composites clearly have macropores ranging from 0.9 to 25 μm. Fe–Co/NC and rGO are connected, which enhances the stability of the composites. As shown in Fig. S3, the Co, Fe, and N evenly locate whole composite. The internal microstructures of the hierarchically porous Fe–Co/NC/rGO were further explored by HRTEM. In Fig. 2e, the porous structure of Fe–Co/NC and typical wrinkled sheets of rGO can be clearly observed. In Fig. 2f, the clear lattice demonstrates the good crystallinity of the Fe–Co alloy for the Fe–Co/NC/rGO, in which the lattice spacing of 0.208 nm is in accord with the (110) crystal plane of Fe–Co alloy. The Fe–Co alloy nanoparticles are surrounded by the graphitized NC layer. This unique structure is conducive to good electromagnetic matching.

**Fig. 3:**
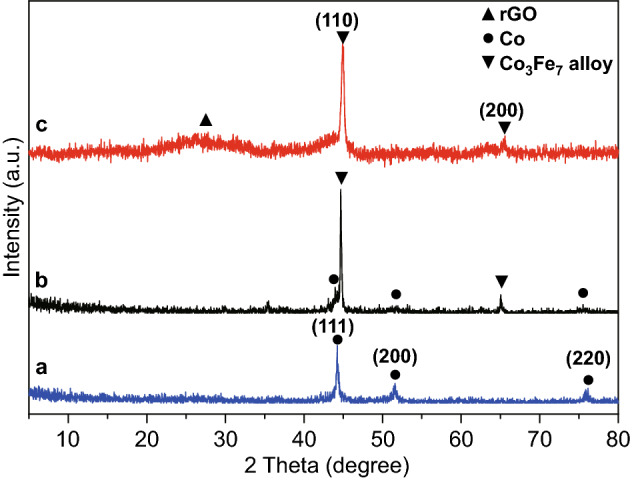
XRD patterns of **a** Co/NC, **b** Fe–Co/NC, and **c** Fe–Co/NC/rGO

The XRD patterns were employed to analyze the phase and structures of the products. As shown in Fig. 3a, three characteristic diffraction peaks were obtained at 2*θ* = 44.2°, 51.5°, and 75.9° for Co/NC, which can be attributed to the (111), (200), and (220) crystal planes of body-centered cubic Co metal (JCPDS No. 15-0806) [38]. This suggests that the Co^2+^ in the precursor was successfully reduced to metal particles. In Fig. 3b, there are two main diffraction peaks in Fe–Co/NC, which correspond to the (110) and (200) crystal planes of Co_3_Fe_7_ alloy (JCPDS No. 48-1816). The result reveals that the Fe ion is embedded in the Co-MOF, and the Fe–Co alloy is formed via carbonization. There are also some peaks of Co, indicating the coexistence of Co_3_Fe_7_ and Co. As shown in Fig. 3c, the main diffraction peaks of Fe–Co/NC/rGO are basically consistent with those of Fe–Co/NC, except that a weak wide peak attributed to rGO appears at approximately 26°.

**Fig. 4:**
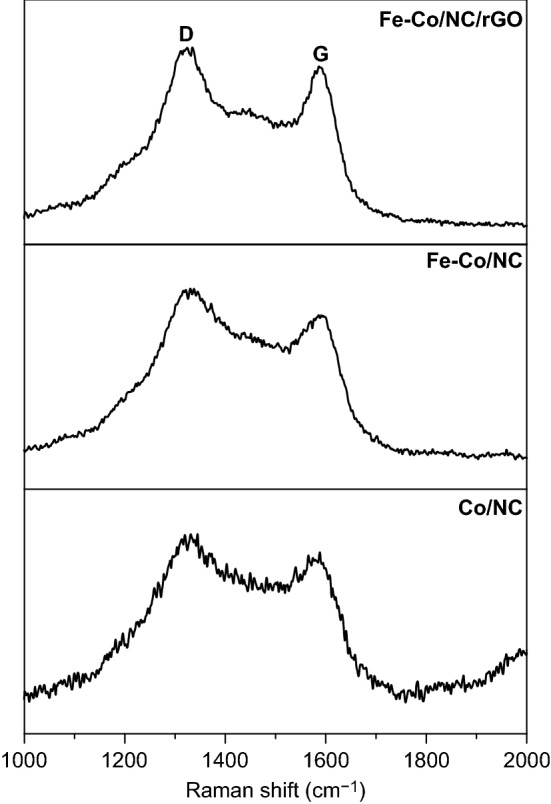
Raman spectra of Co/NC, Fe–Co/NC, and Fe–Co/NC/rGO

The carbon structure of the samples was detected by Raman spectroscopy. As shown in Fig. 4, the three samples all exhibit two peaks at approximately 1330 and 1590 cm^−1^, which can be assigned to the typical D and G bands of carbon materials. The D band usually reflects the lattice defects and disorder degree of carbon materials. The G band indicates the graphitic degree of carbon atoms [39, 40]. The intensity ratio of the D and G bands (*I*_D_/*I*_G_) is a common criterion for evaluating the degree of graphitization of carbon materials. In Fig. 4, the *I*_D_/*I*_G_ of Co/NC, Fe–Co/NC, and Fe–Co/NC/rGO is 1.06, 1.10, and 1.09, respectively. The intensity of D bands is stronger than that of G bands (*I*_D_/*I*_G_ > 1) for all samples, indicating that there are many defects in the samples. This is due to the introduction of N atoms into the carbon skeleton. These defects can serve as a polarization center to further elevate the dielectric loss. Under the premise of impedance matching, the increase in dielectric loss due to the defects is beneficial to improve the absorbing performances [40, 41].

**Fig. 5:**
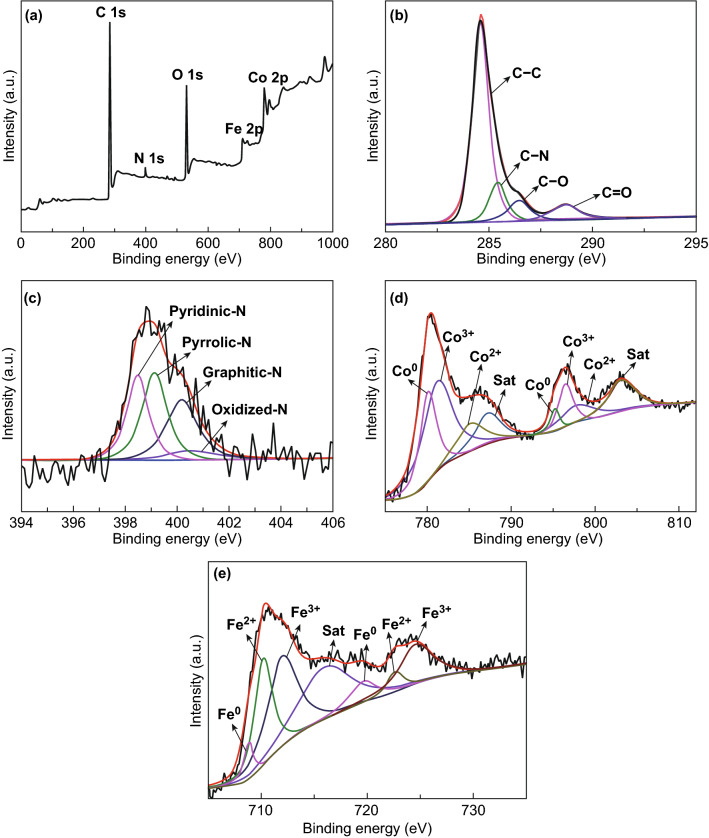
XPS spectra of Fe–Co/NC/rGO of **a** survey scan, **b** C 1s, **c** N 1s, **d** Co 2p, and **e** Fe 2p

The chemical composition and valence state of Fe–Co/NC/rGO were studied by XPS. Based on XPS, the nitrogen content of Fe–Co/NC/rGO is 3.51 at.%. Figure 5a reveals that Fe–Co/NC/rGO consists of C, N, O, Co, and Fe elements. In Fig. 5b, the four peaks in the C 1s spectra correspond to C–C (284.6 eV), C–N (285.2 eV), C–O (286.5 eV), and C=O (288.7 eV). The peak intensities of O1s, C–O, and C=O are weak, illustrating that most oxygen-containing functional groups disappeared through the reduction process. As shown in Fig. 5c, the N 1s spectra are well divided into four peaks, which can be assigned to primary pyridine N (398.1 eV), pyrrole N (398.9 eV), graphite N (399.9 eV), and oxidized N (400.8 eV). This demonstrates that the N atoms have been doped into the carbon lattice to form defects, which could act as dipoles to enhance the dielectric loss. In Fig. 5d, the Co 2p spectra are fitted to three Co states; peaks at 780.1 and 795.2 eV belong to Co^0^, peaks at 785.2 and 797.8 eV are ascribed to Co^2+^, and peaks at 781.2 and 796.5 eV are attributed to Co^3+^. The satellite peaks are located at 787.2 and 803.1 eV. The multiple valence states of Co suggest the surface oxidation of Co NPs in air [40, 42]. As shown in Fig. 5e, Fe 2p of the composite can be decomposed into Fe^0^, Fe^2+^, and Fe^3+^. The peaks with binding energies of 708.8 and 719.9 eV are indexed to Fe^0^, and 710.2 and 722.6 eV are Fe^2+^, while 712.1 and 724.6 eV correspond to Fe^3+^ [43–46].

The TG curves are shown in Fig. S3. The weight percentages of metal for Co/NC, Fe–Co/NC, and Fe–Co/NC/rGO can be estimated to be 42.5, 58.2, and 41.0 wt%, respectively, from the weight loss in the thermogravimetric curves obtained in flowing air (60 mL min^−1^) using Eq. 1.

1 $$C \left( {{\text{wt}}\% } \right) = \left( {\frac{{m_{r} }}{{m_{i} }}} \right) *2 *A_{\text{M}} /M_{{{\text{M}}_{2} {\text{O}}_{3} }}$$

where * C* (wt%) is the content of metal, *m*_r_ is the remaining weight, *m*_i_ is the initial weight of the sample, *A*_M_ is the atomic weight of metal, and *M*_M2O3_ is the molecular weight of M_2_O_3_. In the case of Fe–Co/NC and Fe–Co/NC/rGO, the average atomic weight of the Fe–Co alloy and the average molecular weight of the corresponding oxides are calculated based on the initial Fe/Co molar ratios of the preparation process for Fe-doped Co-MOF and used in Eq. 1.

**Fig. 6:**
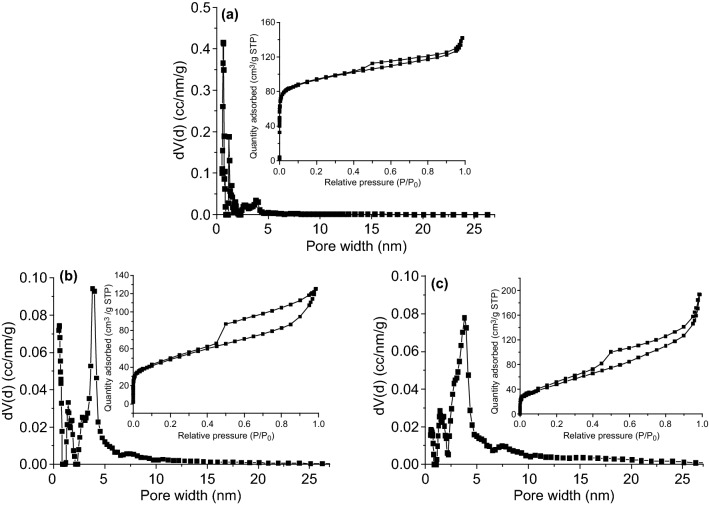
Pore size distribution plots and N_2_ adsorption–desorption isotherms of **a** Co/NC, **b** Fe–Co/NC, and **c** Fe–Co/NC/rGO

The nitrogen adsorption–desorption isotherms are used to analyze the pore structure of the samples. In Fig. 6, Co/NC, Fe–Co/NC, and Fe–Co/NC/rGO exhibit the typical type IV isotherm [47]. The further pore structure of the samples can be observed in the pore size distribution based on a nonlocal density functional theory (NLDFT) model. In Fig. 6a, the pore size distribution of Co/NC profiles has peaks in the range of 0.4–4.4 nm. In Fig. 6b, Fe–Co/NC shows a pore size distribution of 0.4–15.4 nm. This indicates that MOF-derived Co/NC and Fe–Co/NC are materials with both micropores and mesopores. However, Fe doping broadens the pore size distribution. In addition, the pore volume of Fe–Co/NC is 0.176 mL g^−1^, which is smaller than the 0.187 mL g^−1^ of Co/NC. This demonstrates that Fe doping decreases the porosity of the material. As shown in Fig. 6c, the pore sizes of Fe–Co/NC/rGO are in the range of 0.4–25.0 nm, including micropores (0.4–2.0 nm) and mesopores (2.0–25.0 nm). Combining the SEM image and pore size distribution, macropores, mesopores, and micropores are observed to coexist in Fe–Co/NC/rGO composites. In other words, the Fe–Co/NC/rGO composite shows a hierarchically porous structure.

### Magnetic Properties

**Fig. 7:**
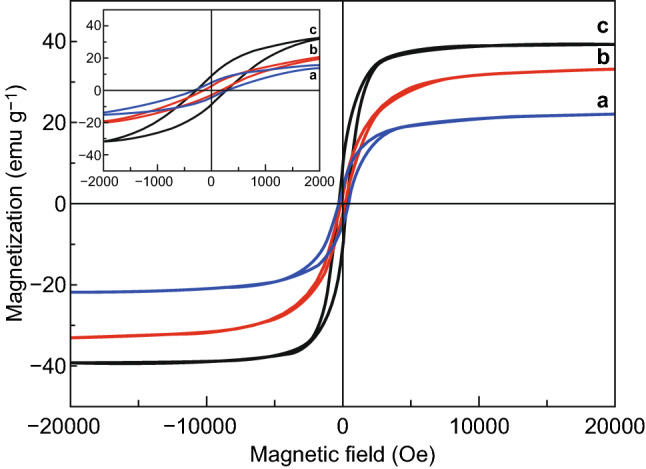
Magnetic hysteresis loops for **a** Fe–Co/NC/rGO, **b** Co/NC, and **c** Fe–Co/NC

The magnetic hysteresis loops of Co/NC, Fe–Co/NC, and Fe–Co/NC/rGO composites are shown in Fig. 7. Compared to the saturation magnetization (*M*s) of 32.96 emu g^−1^ for Co/NC (Fig. 7b), the *M*s of Fe–Co/NC increased to 39.30 emu g^−1^ (Fig. 7c), which proves that Fe doping improves the magnetic properties of the materials. As shown in Fig. 7a, the *M*s of Fe–Co/NC/rGO is 22.50 emu g^−1^ due to the introduction of nonmagnetic rGO. The coercivity of Co/NC is 139.63 Oe, which is lower than those of Fe–Co/NC and Fe–Co/NC/rGO (270.90 and 300.16 Oe, respectively).

### Electromagnetic Parameters and Microwave Absorption Performance

**Fig. 8:**
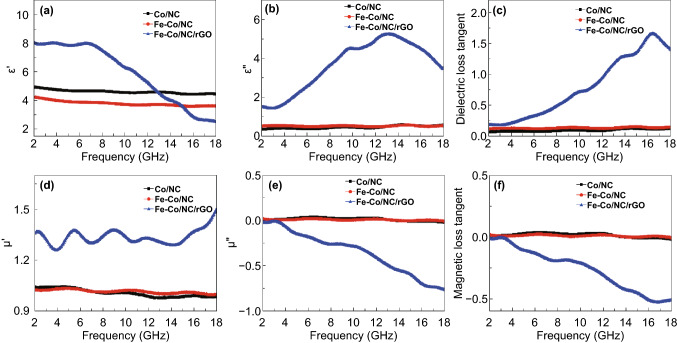
**a** Permittivity real part $\varepsilon^{\prime}$, **b** permittivity imaginary part $\varepsilon^{\prime\prime}$, **c** dielectric loss tangent tan*δ*_e_, **d** permeability real part $\mu^{\prime}$, **e** permeability imaginary part $\mu^{\prime\prime}$, and **f** magnetic loss tangent tan*δ*_m_ of Co/NC, Fe–Co/NC, and Fe–Co/NC/rGO

The electromagnetic parameters are used to evaluate the microwave absorbing properties. Figure 8 gives the relative complex permittivity ($\varepsilon_{r}$ = $\varepsilon^{\prime}$ − $j\varepsilon^{\prime\prime}$), dielectric loss tangent (tan*δ*_e_ = $\varepsilon^{\prime\prime}$/$\varepsilon^{\prime}$), relative complex permeability ($\mu_{r}$ = $\mu^{\prime}$ − $j\mu^{\prime\prime}$), and the magnetic loss tangent (tan*δ*_m_ = $\mu^{\prime\prime}$/$\mu^{\prime}$). The real parts of the complex permittivity ($\varepsilon^{\prime}$) and permeability ($\mu^{\prime}$) stand for the storage ability for electrical and magnetic energy, whereas the imaginary parts ($\varepsilon^{\prime\prime}$ and $\mu^{\prime\prime}$) are related to the dissipation ability [48]. As shown in Fig. 8a, b, $\varepsilon^{\prime}$ and $\varepsilon^{\prime\prime}$ of the Fe–Co/NC/rGO composite are larger than those of Co/NC and Fe–Co/NC. In the range of 12.8–18 GHz, $\varepsilon^{\prime}$ of the composite is smaller than that of Co-NC or Fe–Co/NC, while $\varepsilon^{\prime\prime}$ of the composite is still large, resulting in greater dielectric loss. In Fig. 8c, tan*δ*_e_ of Fe–Co/NC/rGO is much higher than that of the other two samples due to the introduction of rGO; rich defects on rGO and NC and the interfacial polarization enhance the dielectric loss of the composite [49]. As shown in Fig. 8d–f, $\mu^{\prime}$ of the Fe–Co/NC/rGO is larger than that of Co/NC and Fe–Co/NC, $\mu^{\prime\prime}$ and tan*δ*_m_ of Fe–Co/NC/rGO are smaller than those of other two samples. The introduction of nonmagnetic rGO leads to the decrease in the magnetic loss of the composite.

The microwave absorption performances are evaluated by the RL_min_, which is calculated via the electromagnetic parameters [50].

2 $${\text{RL}}\left( {\text{dB}} \right) = 20\log \left|\frac{{z_{\text{in}} - z_{0} }}{{z_{\text{in}} + z_{0} }}\right|$$

3 $$z_{\text{in}} = z_{0} \sqrt {\frac{{\mu_{\text{r}} }}{{\varepsilon_{\text{r}} }}} \tan h\left( {j\frac{2\pi fd}{c}\sqrt {\mu_{\text{r}} \varepsilon_{\text{r}} } } \right)$$

where $z_{0}$ is the characteristic impedance of the free space, $z_{\text{in}}$ is the input impedance, and $\varepsilon_{\text{r}}$ and $\mu_{\text{r}}$ are the relative complex permittivity and permeability. *f* is the frequency of microwave, *d* is the thickness of the absorber, and *c* is the velocity of light.

**Fig. 9:**
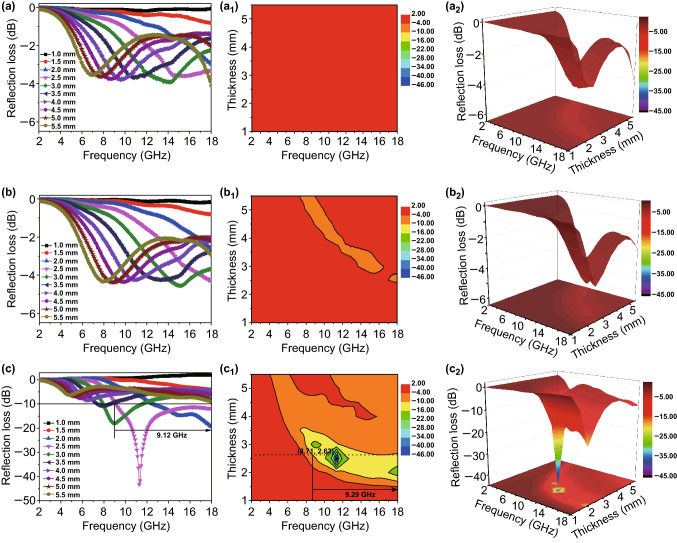
Calculated 2D/3D reflection loss of Co/NC (**a**, **a**_**2**_), Fe–Co/NC (**b**, **b**_**2**_), Fe–Co/NC/rGO (**c**, **c**_**2**_) and contour maps of the reflection loss of Co/NC (**a**_**1**_), Fe–Co/NC (**b**_**1**_), and Fe–Co/NC/rGO (**c**_**1**_) with the mass filling ratio of 25 wt%

Figure 9 reveals the microwave absorption performances of Co/NC, Fe–Co/NC, and Fe–Co/NC/rGO with the loading of 25 wt%. In Fig. 9a (a_1_ and a_2_), none of the RL_min_ of Co/NC exceeds − 10 dB in the whole frequency and thickness ranges. In Fig. 9b (b_1_ and b_2_), although the RL_min_ of Fe–Co/NC is slightly higher than that of Co/NC, it does not yet reach the effective absorption (*RL *< − 10 dB). This shows that neither of the two materials can achieve effective absorption. As shown in Fig. S5, $\mu^{\prime}$ of the Fe–Co/NC is larger than that of Co/NC in the range of 5–17.5 GHz, and $\mu^{\prime\prime}$ of Fe–Co/NC is larger than that of Co/NC in 12.5–18 GHz. In Fig. S6, when the mass filling ratios of the materials reach 55 wt%, their microwave absorption performance is enhanced. The RL_min_ of Fe–Co/NC is − 21.83 dB, and the effective bandwidth is 4.39 GHz. Although the RL_min_ of Co/NC is also improved (from − 4.09 to − 10.63 dB), its microwave absorption performance is still much worse than that of Fe–Co/NC. This illustrates that Fe-doped Co/NC is beneficial to enhance the absorption properties of materials. In Fig. 9c (c_1_ and c_2_), the microwave absorption performance of Fe–Co/NC/rGO composite is greatly improved. The RL_min_ reaches − 43.26 dB at 11.28 GHz at a thickness of 2.5 mm, and the effective absorption bandwidth is 9.12 GHz (8.88–18 GHz). Furthermore, in Fig. 9c_2_, the effective bandwidth of the composite achieves 9.29 GHz at the thickness 2.63 mm. In Fig. S7, the RL_min_ of Co/NC/rGO is − 26.14 dB at a thickness of 2.5 mm and the effective bandwidth is 4.4 GHz. The absorption performances of Fe–Co/NC/rGO are much better than those of Co/NC/rGO. The effective bandwidth of the Fe–Co/NC/rGO is much better than that of most absorbers. The result suggests that the Fe–Co/NC/rGO not only enhances the reflection loss but also effectively broadens the absorption bandwidth, which gives it broad application prospects in the research field of absorbing materials.

**Fig. 10:**
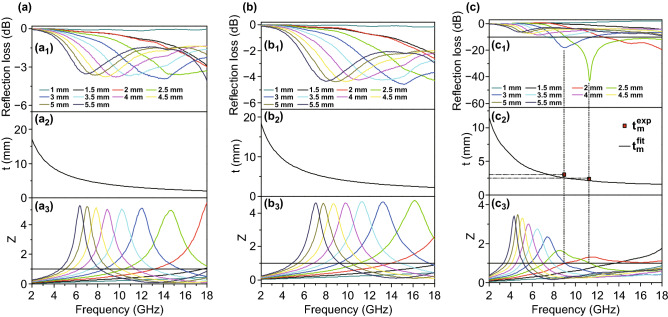
Reflection loss (**a**_**1**_–**c**_**1**_), simulation of the matching thickness ($t_{\text{m}}$) under n = 1 (**a**_**2**_–**c**_**2**_), the modulus of the normalized characteristic impedance matching (Z = |Z_in_/Z_0_|) (**a**_**3**_–**c**_**3**_) for Co/NC (**a**), Fe–Co/NC (**b**), and Fe–Co/NC/rGO (**c**)

According to the microwave absorption mechanism, the excellent absorbing performance of the Fe–Co/NC/rGO benefits from good impedance matching. When *Z* = |*Z*_in_/*Z*_0_| is equal or close to 1, zero reflection appears at the surface of the materials; in other words, the absorbers accomplish a good impedance match. Hence, in order to inquire into the microwave absorption mechanism, the impedance matching characteristics are carefully analyzed in Fig. 10. As shown in Fig. 10a (a_3_), b (b_3_), the values of *Z* of Co/NC and Fe–Co/NC are much higher than 1 at all thicknesses. This impedance mismatch leads to poor absorption. In Fig. 10c (c_3_), the values of *Z* of the Fe–Co/NC/rGO are still far greater than 1 at most thicknesses, and it does not reach effective absorption at these thicknesses. However, at the thicknesses of 2.5 and 3 mm, the values of *Z* are closer to 1, and the absorption properties are enhanced, which confirms that excellent microwave absorption performances are attributed to the good impedance match. In addition, the good impedance match results from the appropriate mass filling ratio, dielectric loss, and magnetic loss. Remarkably, RL_min_ moves to low frequency with the increase in thickness. This phenomenon can be explained by the quarter-wavelength matching model. In the model, the peak frequency ($f_{m}$) and the absorber thickness ($t_{m}$) can be described by Eq. 4 [51, 52].

4 $$t_{\text{m}} = nc/\left( {4f_{\text{m}} \sqrt {\left| {\varepsilon_{\text{r}} } \right|\left| {\mu_{\text{r}} } \right|} } \right),\;\left( {n = 1, \, 3, \, 5, \ldots } \right)$$

When $t_{\text{m}}$ and $f_{\text{m}}$ conform to this equation, incident and reflected microwaves would be out of phase of 180°, resulting in the disappearance of each at the air–absorber interface [53]. As shown in Fig. 10c, the $t_{\text{m}}$(*t*_m_^fit^) curve of Fe–Co/NC/rGO is simulated with Eq. 4, and the matching thicknesses (*t*_m_^exp^) marked with red squares are obtained from the RL_min_ curves. Apparently, the experimental results are consistent with the calculated results at the thicknesses of 2.5 and 3 mm, which indicates that the RL_min_ curves of Fe–Co/NC/rGO conform to the quarter-wavelength matching model. Therefore, the Fe–Co/NC/rGO composite shows excellent microwave absorption performance.

The microwave attenuation constant (*α*) is another crucial evaluation criterion for the microwave absorption performance of an excellent absorber; it can be calculated using Eq. 5 [54–56].

5 $$\upalpha = \frac{{\sqrt {2\pi f} }}{c} \times \sqrt {\left( {\mu^{\prime\prime}\varepsilon^{\prime\prime} - \mu^{\prime}\varepsilon^{\prime}} \right) + \sqrt {\left( {\mu^{\prime\prime}\varepsilon^{\prime\prime} - \mu^{\prime}\varepsilon^{\prime}} \right)^{2} + \left( {\varepsilon^{\prime}\mu^{\prime\prime} + \varepsilon^{\prime\prime}\mu^{\prime}} \right)^{2} } }$$

**Fig. 11:**
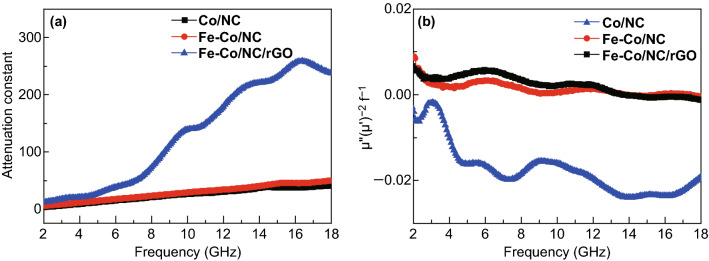
**a** Attenuation constant and **b** plots of $\mu^{\prime\prime}\left( {\mu '} \right)^{ - 2} f^{ - 1}$ of Co/NC, Fe–Co/NC, and Fe–Co/NC/rGO

In Fig. 11a, *α* values of Fe–Co/NC/rGO are the largest among the three samples over the whole frequency range, especially in the high-frequency region. Consequently, Fe–Co/NC/rGO, with a larger loss tangent and the highest attenuation property, exhibits the best microwave absorption performance.

The eddy current loss can be calculated using the formula of $\mu^{\prime\prime}\left( {\mu '} \right)^{ - 2} f^{ - 1} = 2\varPi \mu_{0} \sigma d^{2} /3$, where $\mu_{0}$ is the vacuum permeability, $\sigma$ is the conductivity, and d is the sample thickness. If the magnetic loss only originates from the eddy current loss, then the values of $\mu^{\prime\prime}\left( {\mu '} \right)^{ - 2} f^{ - 1}$ should be constant when the frequency is changed. As shown in Fig. 11b, the values of $\mu^{\prime\prime}\left( {\mu '} \right)^{ - 2} f^{ - 1}$ fluctuate with frequency, which illustrates that the eddy current loss is weak and the magnetic loss mainly comes from magnetic resonance [57, 58]. Magnetic hysteresis loops in Fig. 7 indicate the existence of hysteresis loss for three kinds of materials. In case of Fe–Co/NC and Fe–Co/NC/rGO, the increased area of magnetic hysteresis loops indicates that Fe doping enhances the hysteresis loss.

**Fig. 12:**
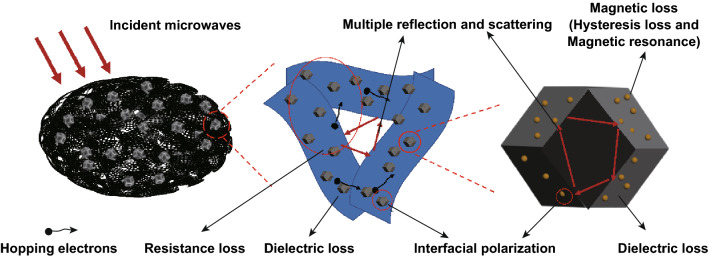
Schematic diagram of microwave absorption mechanism of Fe–Co/NC/rGO

Based on the above discussion, the microwave absorption mechanism of Fe–Co/NC/rGO composite is illustrated in Fig. 12. First, the impedance matching is an important factor affecting the microwave absorption performance of materials [59]. The combination of Fe–Co/NC and rGO gives the composite excellent impedance matching and improves the absorbing performance of the composite. Second, hysteresis loss and magnetic resonance of magnetic metals contribute to the magnetic loss of the composites. Moreover, Fe doping increases the hysteresis loss. On the other hand, Fe–Co alloys embedded in the NC form many interfaces, which increase the polarization center of the material and greatly enhance the interfacial polarization of the material, resulting in the increase in dielectric loss [60, 61]. Third, after carbonization of the Fe–Co-MOF, N-doped C is obtained. The introduction of N atoms into the C lattice and the defects in rGO are beneficial to improve the dielectric loss of the material [62]. In addition, the porous rGO not only enhances dielectric loss but also provides resistance loss. The rGO sheets are overlapped to form a capacitor-like conductive network, which causes the motion of hopping electrons, forming oscillatory current and generating increased resistance loss [63–65]. Moreover, most of the microwaves enter into the absorber due to the good impedance match, and the unique hierarchically porous structure (the macropores, mesopores, and micropores) of Fe–Co/NC/rGO composite then limits more microwaves to the interior of materials. Therefore, the microwaves are reflected and attenuated for many times in the interior of the composite. In another words, the scattering and multiple reflection resulted from the pore structure enhance the microwave absorption [66, 67]. In summary, Fe–Co/NC/rGO with multiple components and a unique hierarchically porous structure exhibits excellent microwave absorption performance.

## Conclusions

A novel lightweight Fe–Co/NC/rGO composite with broadband absorption was prepared using a simple in situ growth method. Fe–Co/NC was uniformly loaded on the sheets of porous cocoon-like rGO. The multiple components and the hierarchically porous structure are responsible for the excellent microwave absorption performances. The RL_min_ is − 43.26 dB with a thickness of 2.5 mm, and the widest effective bandwidth is 9.29 GHz at a thickness of 2.63 mm. The magnetic loss, dielectric loss, resistive loss, good impedance matching, scattering, and multiple reflections contribute to the excellent microwave absorption performance. This study not only provides an excellent absorber but also puts forward a design strategy for lightweight absorbers with broadband absorption.

## Electronic supplementary material

Below is the link to the electronic supplementary material.

Supplementary material 1 (PDF 1122 kb)
