# Supplementary material for: Rational Construction of Hierarchically Porous Fe–Co/N-Doped Carbon/rGO Composites for Broadband Microwave Absorption
Source: Nanomicro Lett. 2019 Sep 13;11:76. doi: 10.1007/s40820-019-0307-8 (PMC7770714; doi:10.1007/s40820-019-0307-8)
Supplement: Supplementary file 1 — Supplementary material 1 (PDF 1122 kb) [file 40820_2019_307_MOESM1_ESM.pdf]

Supporting Information for

Rational Construction of Hierarchically Porous Fe-Co/N-doped Carbon/rGO Composites for Broadband Microwave Absorption

Shanshan Wang[1], Yingchun Xu[1], Ruru Fu[1], Huanhuan Zhu[1], Qingze Jiao[1, 2], Tongying Feng[2], Caihong Feng[1], Daxin Shi[1], Hansheng Li[1], Yun Zhao[1, *]

[1]School of Chemistry and Chemical Engineering, Beijing Institute of Technology, 100081, People's Republic of China

[2] School of Materials and the Environment, Beijing Institute of Technology, Zhuhai, Zhuhai 519085, People's Republic of China

*Corresponding author. E-mail: zhaoyun@bit.edu.cn (Yun Zhao)

Supplementary Figures

Paraffin (0.15 g) was melted by heating. 0.05 g of Co/NC, Fe-Co/NC, or Fe-Co/NC/rGO was added with vigorous stirring. The mixture was then poured into the preheated standard mold. After cooling down, the coaxial ring with outer diameter of 7 mm, inner diameter of 3.04 mm and thickness of 2 mm was obtained.

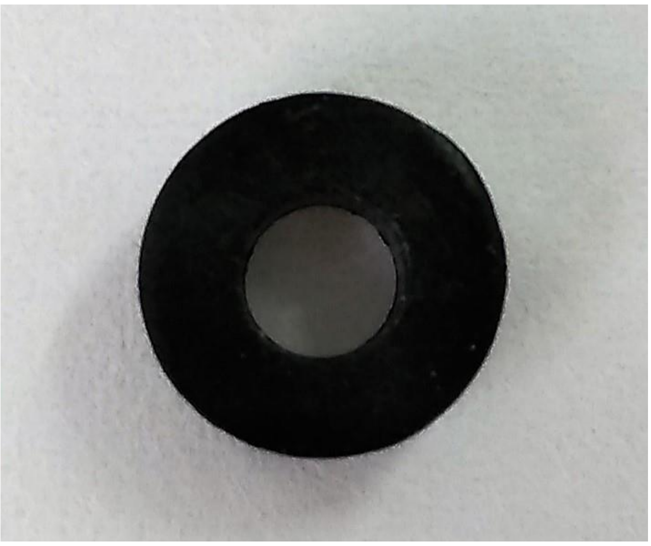

Fig. S1 The coaxial ring of materials with paraffin matrix for measurement of electromagnetic parameters and microwave absorbing performances

The porous cocoon-like rGO was prepared in a four-neck flask with a mechanical stirring. Briefly, 30 mL GO aqueous dispersion (1 mg mL^{-1}) was sonicated for 30 min. 0.12 g ascorbic acid was then added and sonicated for another 10 min. Subsequently, the mixture was transferred to a four-neck flask and kept at 90 °C for 2 h with continuous stirring. The precipitant was then collected and washed with deionized water. The porous cocoon-like rGO was obtained by a freeze-drying process.

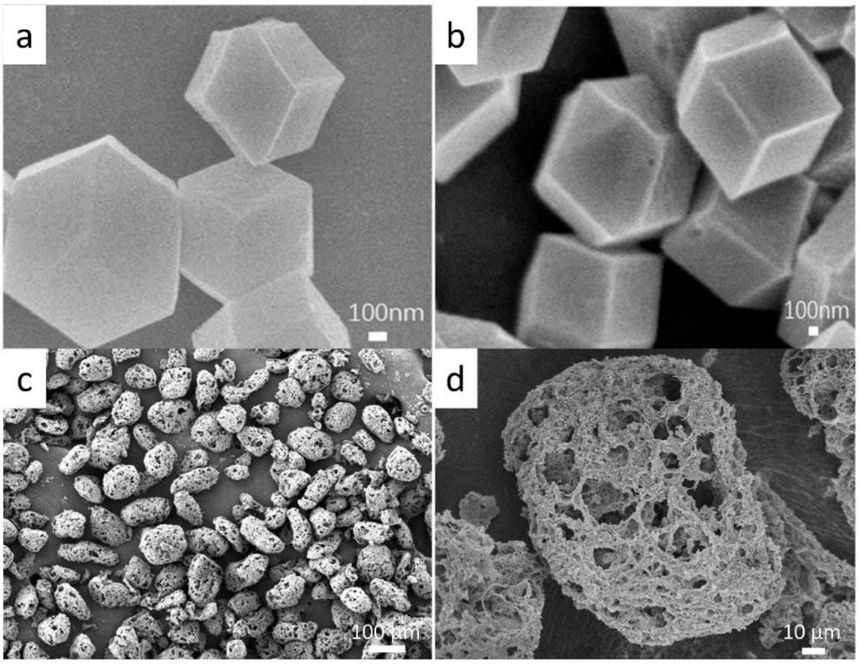

Fig. S2 FESEM images of **a** Co-MOF, **b** Fe doped Co-MOF, **c** porous rGO, and **d** Fe-Co/NC/rGO

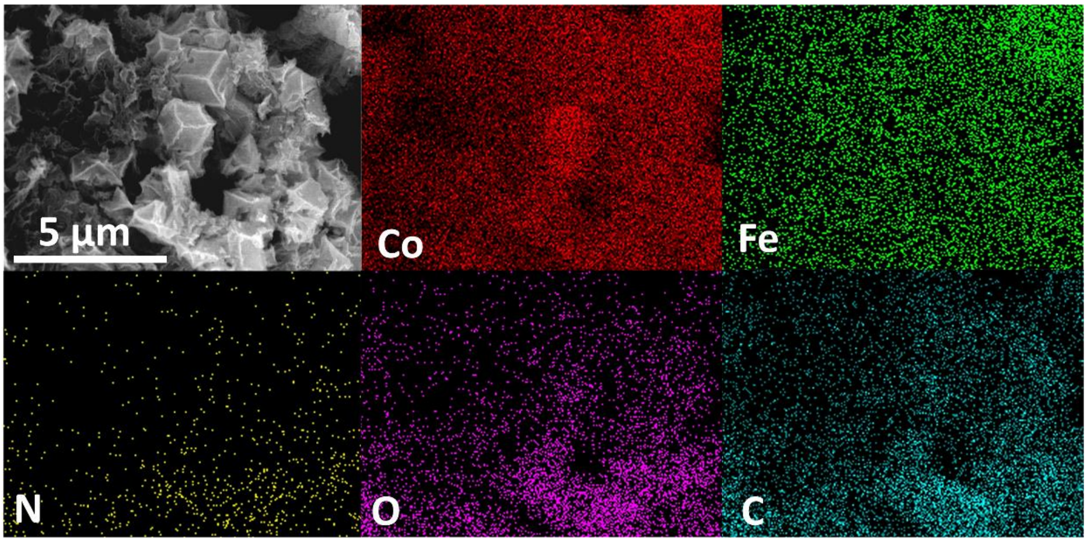

Fig. S3 Elemental mapping of Fe-Co/NC/rGO

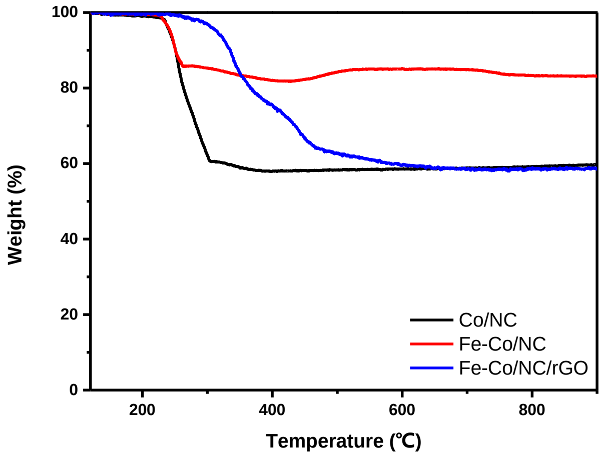

Fig. S4 TG curves of Co/NC, Fe-Co/NC, and Fe-Co/NC/rGO composites in air

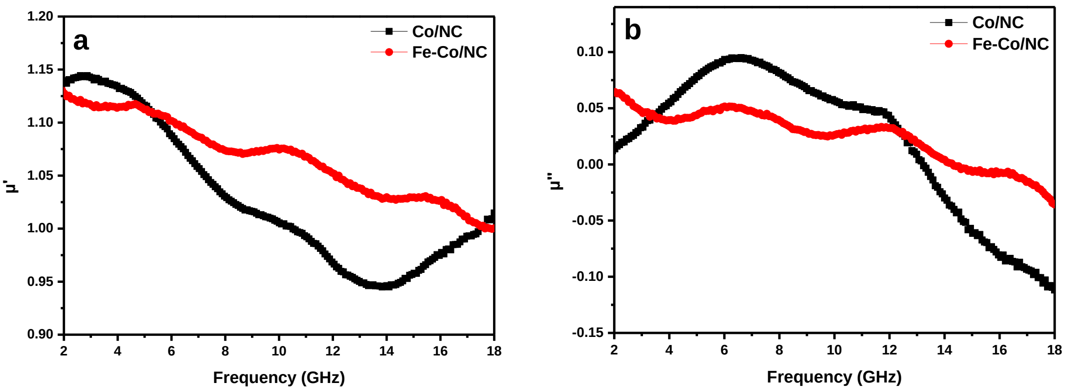

Fig. S5 **a** Permeability real part μ', **b** permeability imaginary part μ'' of Co/NC and Fe-Co/NC at the loading of 55 wt%

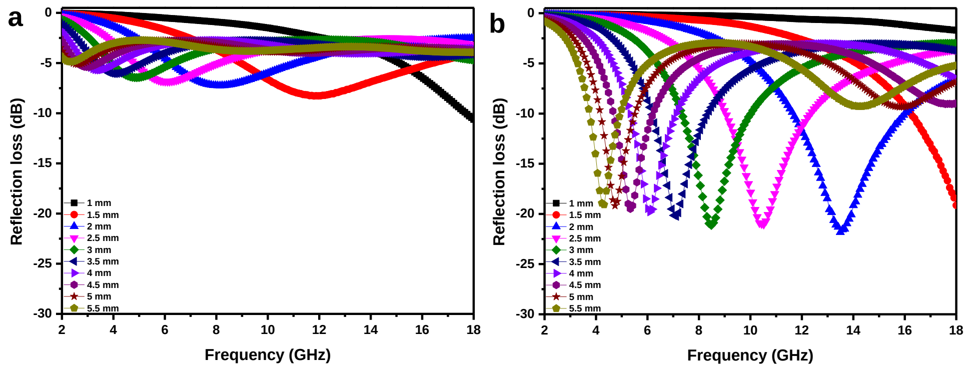

Fig. S6 The calculated reflection loss of **a** Co/NC and **b** Fe-Co/NC/rGO with the mass filling ration of 55 wt%

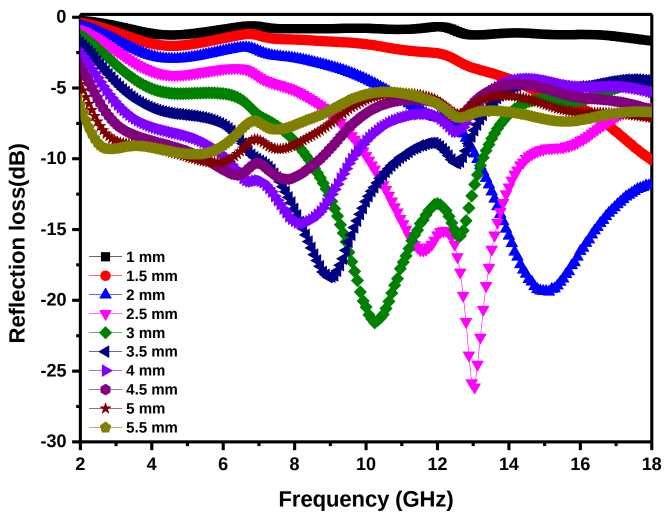

Fig. S7 The reflection loss of Co/NC/rGO

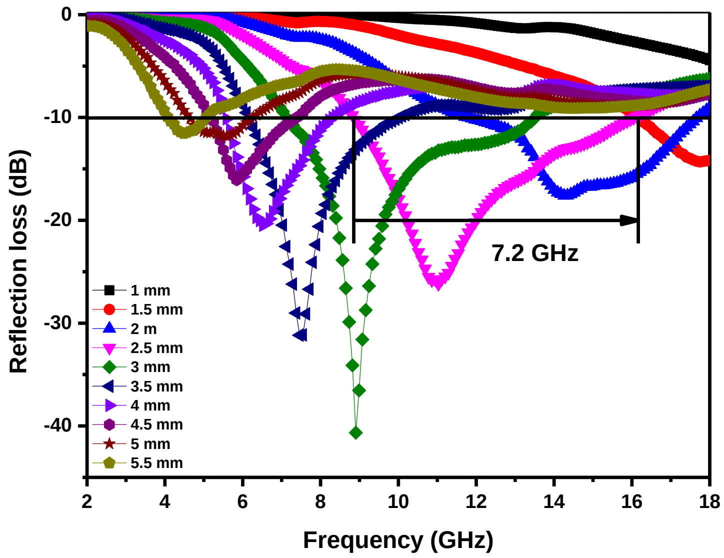

Fig. S8 The reflection loss of Co-Fe/NC/rGO retested

The magnetic behavior may be modulated by the dielectric behavior, which may lead to the coupling between these two parameters. The imaginary parts (ε'' and μ'') represent the loss of electric and magnetic energy. The relationship between ε'' and μ'' can be expressed by the equivalent circuit model of the Fe-Co/NC/rGO composites as shown in Fig. S9. From Fig. 8, the changing trend of the imaginary parts of permeability is just inverse to that of the permittivity, which is attributed to the capacitance *C* lead or lag behind the angle of 90° than the inductance *L*. According to the Maxwell equations, a magnetic field can be induced by an ac electric field and be radiated out. So the negative value denotes that the magnetic energy is

radiated out from the composites and transferred into the electric energy, which can greatly increase ε'' and then leads to the negative μ'' [S1, S2].

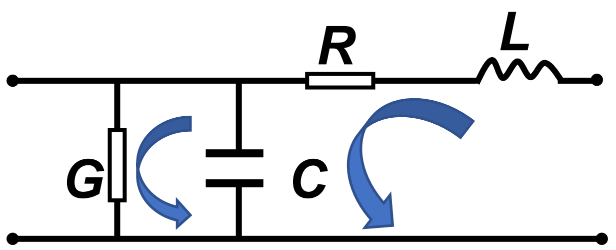

Fig. S9 Equivalent circuit model

Supplementary References

[S1] X.L. Shi, M.S. Cao, J. Yuan, X.Y. Fang, Dual nonlinear dielectric resonance and nesting microwave absorption peaks of hollow cobalt nanochains composites with negative permeability. Appl. Phys. Lett. **95**(16), 163108 (2009). https://doi.org/10.1063/1.3250170

[S2] L. Deng, M. Han, Microwave absorbing performances of multiwalled carbon nanotube composites with negative permeability. Appl. Phys. Lett. **91**(2), 023119 (2007). https://doi.org/10.1063/1.2755875
